# Role of the Community Health Representatives in Caring for Older People in Tribal Communities

**DOI:** 10.1093/geroni/igaf122.389

**Published:** 2025-12-31

**Authors:** Susan Chapman, Angela “Jackie” Kaslow

**Affiliations:** University of California, San Francisco, San Francisco, California, United States; University of California, San Francisco, San Francisco, California, United States

## Abstract

The Community Health Representative (CHR) Program was established 57 years ago to meet the healthcare needs of American Indian and Alaska Native (AIAN) populations. The purpose of this project was to assess whether the CHR workforce in Tribal rural and urban settings had a sufficient supply of well-trained CHRs and resources to meet the health care needs of older people. Twenty (20) individuals were identified from 10 sites in California using snowball sampling. Fourteen (14) participants including 9 CHRs, 2 nurses, 2 managers and 1 clinic director agreed to participate in a 45-60 virtual semi-structured interview. We conducted a sub-thematic analysis focused on issues in caring for older people in rural tribal settings including the unique care needs, barriers to care, and the role of CHRs in meeting those needs. Home visits stood out as an essential service for rural-living older tribal members. Transportation was a highly used and valued service provided by CHRs. Older Tribal people experience social isolation due to remote living, which is also characterized by limited information technology connectivity resulting in limited health technology literacy. Older Tribal people also demonstrate a significant need for transportation to clinical appointments and to obtain medications and medical supplies. Interviewees noted that building trusting relationships that reflect AIAN cultural standards is a key component of older tribal people and acceptance of care. Policy should address the structural gaps to AIAN older people in accessing culturally-based home care services from well-trained CHRs as well as barriers to essential services.

